# The Impact of Diabetes and Edentulism on All-Cause Mortality: Racial and Ethnic Disparities

**DOI:** 10.1093/geroni/igab046.792

**Published:** 2021-12-17

**Authors:** Chenxin Tan, Huabin Luo, Frank Sloan, Brenda Plassman, Samrachana Adhikari, Mark Schwartz, Xiang Qi, Bei Wu

**Affiliations:** 1 New York University, New York, New York, United States; 2 East Carolina University, Greenville, North Carolina, United States; 3 Duke University, Durham, North Carolina, United States; 4 New York University, NYC, New York, United States; 5 Rory Meyers College of Nursing, New York, New York, United States

## Abstract

This study examined the relationships between the concomitance of diabetes mellitus (DM) and edentulism and mortality among Black, Hispanic, and White older adults in the US. We used data from the 2006-2016 Health and Retirement Study with 2,108 Black, 1,331 Hispanic, and 11,544 White respondents aged 50+. Results of weighted Cox proportional hazards models showed that the concomitance of DM and edentulism was associated with a higher mortality risk for Blacks (Hazard Ratio [HR] = 1.58, p < 0.01), Hispanics (HR = 2.16, p < 0.001) and Whites (HR = 1.61, p < 0.001). Findings also indicated that DM was a risk factor for mortality across all racial/ethnic groups, but edentulism was a risk factor only for Whites (HR = 1.30, p < 0.001). This study revealed that the risk of DM and edentulism on mortality varied among racial/ethnic groups. Our study gives alternative explanations for the observed findings.

